# Hereditary leiomyomatosis and renal cell cancer (HLRCC), pheochromocytoma (PCC)/paraganglioma (PGL) and germline fumarate hydratase* (FH)* variants

**DOI:** 10.1530/EDM-24-0073

**Published:** 2024-12-20

**Authors:** John J Orrego, Joseph A Chorny

**Affiliations:** ^1^ Department of Endocrinology and Metabolism, Kaiser Foundation Health Plan of Colorado, Denver, Colorado, USA; ^2^ Department of Pathology, Kaiser Foundation Health Plan of Colorado, Denver, Colorado, USA

**Keywords:** endocrine cancers, adrenal, rare diseases/syndromes

## Abstract

**Summary:**

Hereditary leiomyomatosis and renal cell cancer (HLRCC) is an autosomal dominant condition characterized by multiple cutaneous and uterine leiomyomas and renal cell cancer (RCC). HLRCC is caused by germline pathogenic/likely pathogenic (P/LP) variants in the fumarate hydratase* (FH)* gene on chromosome 1q42.3, encoding the mitochondrial enzyme responsible for the conversion of fumarate to malate in the Krebs cycle. 0.6–3.1% of individuals with pheochromocytoma/paraganglioma (PCC/PGL) carry a germline variant in the *FH* gene. Most of these patients have no personal or family history of HLRCC-associated manifestations, but some of them do. We described a female-to-male transgender with HLRCC who presented with large symptomatic uterine leiomyomas in the third decade of life and was diagnosed with a PCC 19 years after hysterectomy and with cutaneous leiomyomas and an aggressive form of RCC in the sixth decade of life. With the publication of this case and the review of the existent literature, and until more information becomes available, we would like to emphasize that clinicians should be aware of the possible connection between HLRCC and PCC/PGL, that genetic testing for susceptibly genes for PCC/PGL should include the *FH* gene and finally that patients with HLRCC should be screened for PCC/PGL.

**Learning points:**

- HLRCC, an autosomal dominant condition caused by germline P/LP variants in the fumarate hydratase* (FH)* gene, is characterized by multiple cutaneous and uterine leiomyomas and RCC.
- 0.6–3.1% of individuals with PCC/PGL carry a germline P/LP variant in the *FH* gene.
- Most of these patients have no personal or family history of HLRCC-associated manifestations, but some of them do.
- Preliminary evidence suggests that genetic testing for susceptibly genes for PCC/PGL should include the *FH* gene and that patients with HLRCC should be screened for PCC/PGL.
- Until more information becomes available, we suggest doing a full history, physical, family history, and screen for HLRCC-associated manifestations when there is an *FH* variant.
- Screening for PCC/PGL in patients with HLRCC could potentially include a baseline whole-body MRI and plasma fractionated metanephrines.

## Background

Hereditary leiomyomatosis and renal cell cancer (HLRCC) is an autosomal dominant condition characterized by multiple cutaneous and uterine leiomyomas and renal cell cancer (RCC) (1, 2). HLRCC is caused by germline pathogenic/likely pathogenic (P/LP) variants in the fumarate hydratase* (FH)* gene on chromosome 1q42.3, encoding the mitochondrial enzyme responsible for the conversion of fumarate to malate in the Krebs cycle (3). In addition, there is preliminary evidence correlating germline P/LP *FH* variants with hereditary pheochromocytoma/paraganglioma (PCC/PGL) in patients without clinical manifestations of HLRCC. Less frequently, HLRCC individuals present with PCC/PGL, like the patient described here. With the publication of this case and the review of the existent literature, we expect to shed light on the link between germline *FH* variants, HLRCC and PCC/PGL.

We describe a female-to-male transgender with HLRCC, who had a hysterectomy and unilateral oophorectomy at age 28 years for large uterine leiomyomas, a left adrenalectomy at age 47 years for a PCC, multiple piloleiomyomas removed after age 50 years and a right radical nephrectomy at age 55 years for an aggressive RCC with prominent tubulocystic growth pattern.

## Case presentation

A 57-year-old female-to-male transgender on testosterone for 11 years, status post-bilateral mastectomy 9 years ago, was evaluated for adrenal insufficiency.

## Investigation

**Figure 1:**
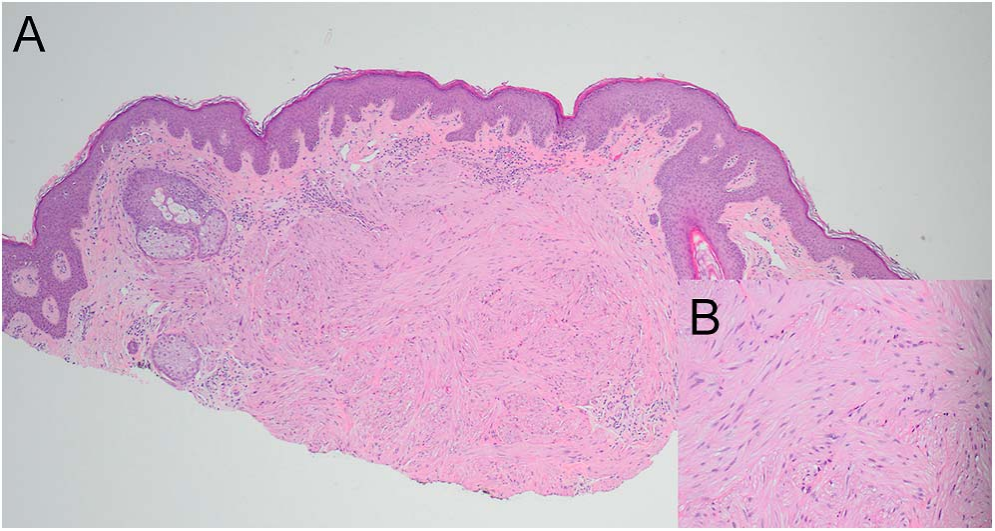
(A) Histopathology: cutaneous piloleiomyoma of the chin. Proliferation of bland and haphazard smooth muscle cells in the dermis (H&E ×40). (B) Histopathology: the inset displays the smooth muscle cells, which lack cytologic atypia or mitotic activity (H&E ×200). As another biopsy from the right arm also revealed a cutaneous leiomyoma, the possibility of HLRCC was raised in the pathology report.

He had a hysterectomy and unilateral salpingo-oophorectomy at age 28 years for large and symptomatic uterine leiomyomas and a left adrenalectomy at age 47 for a PCC of unknown size. Although biochemical and radiological data could not be retrieved, the pathological description of the resected specimen reported a well-circumscribed tumor arising from the adrenal medulla, composed of small compact groups of cells (alveolar and anastomosing trabecular patterns). The nuclei were round with fine chromatin clumping, and the cytoplasm was abundant and slightly granular. Necrosis and mitotic activity were not identified, and the Ki-67 index was not reported. The tumor did not invade through the capsule or into the adjacent adrenal gland. Immunohistochemistry (IHC) showed positivity for CD56 (NCAM), chromogranin and synaptophysin within the tumor and for S100 within the sustentacular cells. Given that the tissue had been discarded after 10 years, we were unable to stain the tumor for *FH* and 2-succinocysteine (2SC) to determine if the PCC was FH-deficient. Full gene sequencing and deletion/duplication analysis did not detect germline variants in the *VHL*, *SDHB*, *SDHC*, *SDHD*, *SDHAF2*, *MAX* and *TMEM127* genes. Plasma fractionated free metanephrines were not measured annually for surveillance as it is recommended for these patients. The patient had a punch biopsy of a right mid back skin nodule at age 50 years and shave biopsies of the left inferior chin and the right upper arm skin lesions at age 55 years that were consistent with piloleiomyomas (Fig. 1).

**Figure 2:**
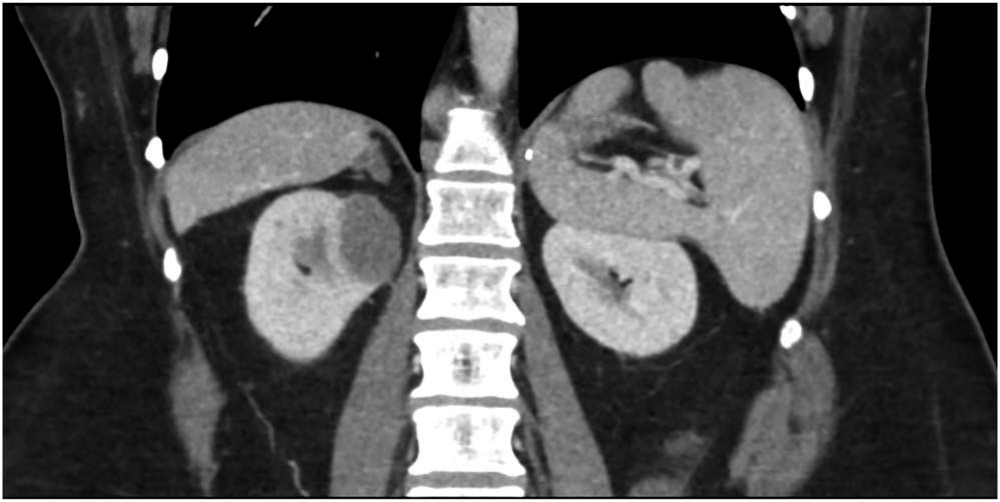
CT of the abdomen and pelvis, performed after the administration of intravenous contrast material, showed a solid 3.2-cm renal mass suspicious for renal abscess vs low-grade papillary/chromophobe RCC.

**Figure 3:**
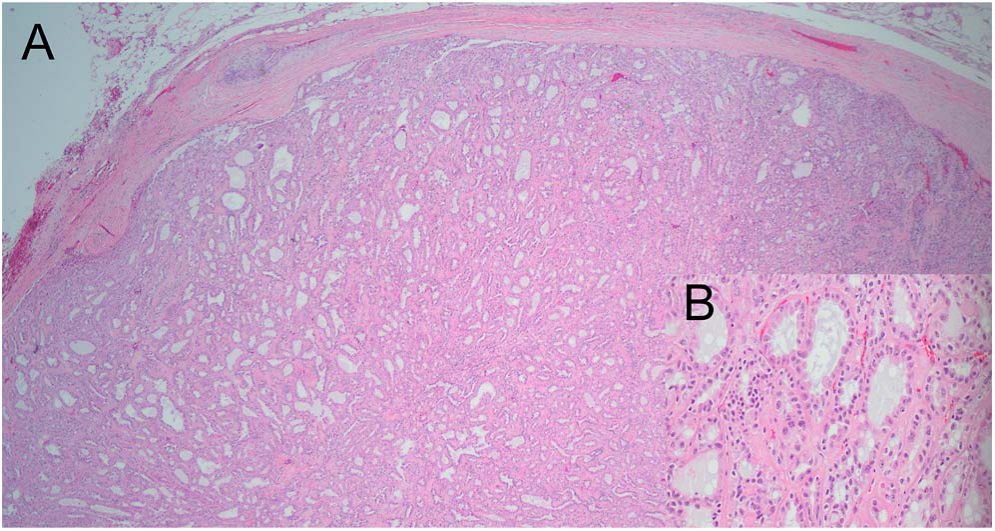
(A) Histopathology: metastatic renal cell carcinoma, ISUP/WHO grade 3, in a perinephric lymph node. The tumor is composed of tubules and cysts resembling a tubulocystic renal cell carcinoma (H&E ×20) but was FH-deficient by immunohistochemistry, indicating a more aggressive tumor. (B) Histopathology: cells with prominent nucleoli and abundant eosinophilic cytoplasm (H&E ×200).

He presented with abdominal pain at age 55 years, and a CT of the abdomen and pelvis, performed with and without contrast, showed a solid 3.2-cm renal mass with associated pericaval and retrocaval adenopathy measuring up to 2 cm (Fig. 2). An MRI of the abdomen, performed with and without gadolinium, showed an upper pole right kidney mass and retroperitoneal adenopathy, felt to be consistent with RCC and nodal metastases. A CT-guided biopsy of the renal mass and a right pericaval lymph node revealed metastatic RCC. IHC showed expression of CA IX, AMACR and PAX8 and was negative for CK7, CD117 and CD10. A chest CT, performed after the administration of contrast material, revealed numerous sub-5 mm noncalcified pulmonary nodules scattered throughout the lungs bilaterally. Right radical nephrectomy, right adrenalectomy and retroperitoneal and supraceliac aortic lymph node dissections were undertaken. Pathological examination of the resected specimen (Fig. 3) disclosed a 4.6-cm RCC with prominent tubulocystic growth pattern, with negative margins, WHO/ISUP grade 3, metastatic to all 16 lymph nodes in the interaortocaval and supraceliac periaortic regions. StrataNGS detected a somatic missense mutation in the *FH* gene, c.1457C>A (p.Ala486Asp), with loss of heterozygosity (due to a single-copy loss). IHC (performed at Mayo Clinic Laboratories, USA) for FH was negative, and there was strong aberrant staining for 2SC consistent with an HLRCC-associated (WHO, 2016) or FH-deficient (GUPS, 2021) RCC. Sequence analysis and deletion/duplication testing of the *FH* gene (Invitae Hereditary Leiomyomatosis and Renal Cell Cancer Test) revealed one heterozygous pathogenic missense variant c.1457C>A (p.Ala486Asp). This variant, previously known as c.1328C>A (p.A443D), had been reported in three family members with HLRCC. The index case, a 35-year-old woman with cutaneous leiomyomatosis since age 14 years, had a subtotal hysterectomy at age 25 years for menorrhagia from uterine leiomyomas. Her first cousin also had multiple cutaneous and uterine leiomyomas necessitating a total hysterectomy. A PET scan of both revealed no occult neoplasms. Her brother, with no leiomyomas on examination, or PCC/PGL on imaging, died from metastatic RCC at age 27 years (4).

Our patient’s mother died at age 30 years old from uterine cancer. His father died at age 62 years old from cardiovascular disease. There was no family history of PCC/PGL or RCC. He had no children.

## Treatment, outcome and follow-up

**Figure 4:**
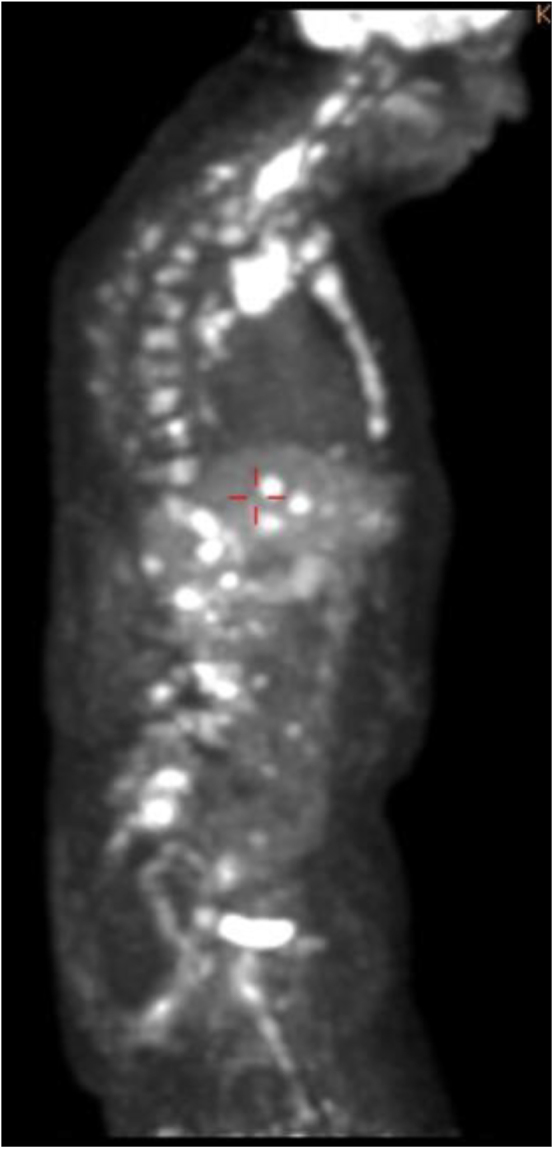
Fludeoxyglucose-18 (FDG) PET scan showed extensive multisystem metastatic disease affecting the lungs, pleura, liver, skeleton and lymph nodes above and below the diaphragm.

Six months later, the patient presented with lower back pain, and an MRI of the lumbar spine without contrast showed multiple osseous metastases in the lower thoracic and lumbar spine. He was treated with erlotinib and bevacizumab and with palliative intent radiation therapy from L1 to L4. Fourteen months later, CT of the chest, abdomen and pelvis, performed with and without contrast, MRI of the spine without contrast and fludeoxyglucose-18 (FDG) PET scan (Fig. 4) showed extensive multisystem metastatic disease affecting the lungs, pleura, liver, skeleton and lymph nodes above and below the diaphragm. The patient denied hyperadrenergic spells and serum plasma total fractionated metanephrines were normal, ruling out metastatic secreting PCC. He was treated with palliative intent radiation therapy from T7 to T11. Erlotinib and bevacizumab were discontinued. Given his rapid decline in performance status and the toxicity profile associated with pazopanib, the patient decided to pursue comfort measures only.

## Discussion

Most patients with HLRCC develop multiple cutaneous leiomyomas at a mean age of 25 years. Eighty percent of women with HLRCC develop multiple and large symptomatic uterine leiomyomas at a mean age of 30 years, and many need a hysterectomy. The lifetime risk of RCC in patients with HLRCC is 15–20%, and the median age at diagnosis is 40 years (5). Most HLRCC-associated RCCs are aggressive and are metastatic at diagnosis. HLRCC-associated RCCs have uncommon growth patterns with papillary architecture being the most common, but other growth patterns include solid, cribriform, tubulocystic and cystic. While the RCC in this case had a pure tubulocystic pattern, it is best classified as a HLRCC-associated or FH-deficient RCC to indicate adverse prognosis. Pure tubulocystic RCCs (WHO, 2016) are rare and, while having grade 3 prominent nucleoli, generally have an indolent prognosis (6).

Studies searching for new susceptibility genes in patients with PCC/PGL identified causal germline P/LP *FH* variants in some of these individuals. Occasional patients with HLRCC develop PCC/PGL. Whether individuals with *FH*-related PCC/PGL are at a high risk of experiencing clinical manifestations of HLRCC or patients with HLRCC have an increased risk of developing PCC/PGL is currently unclear due to the small number of patients reported.

PCC/PGLs belonging to the pseudohypoxic cluster are characterized by activation of pathways that initiate the hypoxia signaling. Cluster 1A associated genes, including *FH*, disrupt the Krebs cycle and result in the impairment of mitochondrial oxidative phosphorylation, leading to severe depletion of ATP synthesis, which needs to be compensated for by increased cellular glycolysis. The alteration of genes of the Krebs cycle results in the accumulation of succinate, fumarate or malate, oncometabolites known to promote DNA hypermethylation and inactivation of tumor suppressor genes, resulting in decreased hypoxia-inducible factor (HIF)-α hydroxylation and reduced HIF-α ubiquitination/degradation. The resultant HIF-α accumulation promotes angiogenesis, tumor extravasation, migration, invasion and metastases (7).

In a PCC/PGL cohort that included 145 patients (8), methylome analysis identified a single tumor carrying no *SDHx* mutation that displayed a hypermethylator phenotype and had an ‘*SDH*-like’ behavior for a prevalence of 0.7%. The tumor was a local recurrence of a PCC resected from a woman presenting with high levels of urinary normetanephrine. Whole-exome sequencing of tumor and matched blood DNA detected a somatic *FH*-inactivating variant, c.1043G>C (p.Gly348Ala), and a pathogenic germline *FH* variant, c.349G>C (p.Ala117Pro), respectively (8). She had undergone a total hysterectomy at age 35 years for hemorrhagic fibromas. This germline mutation had been previously described in patients with multiple leiomyomatosis and/or RCC, displaying reduced FH activity (8).

The same group screened 598 PCC/PGL patients, without germline P/LP variants in 11 known PCC/PGL susceptibility genes, for germline *FH* variants and large deletions and identified five pathogenic variants in five patients (0.8%) (9), including the patient reported above. Excluding this individual, two of the remaining four patients had both PCCs and PGLs and one patient each had a single and multiple PGLs, respectively. Two patients developed metastatic disease. None of these four patients had RCC or HLRCC manifestations (9).

Clark and coworkers found that among the 71 patients with PCC/PGL, two individuals had pathogenic germline *FH* variants (2.8%). Both patients had unilateral PCCs and did not develop metastatic disease (10). They did not have personal or family history of HLRCC or PCC/PGL. One of these *FH* variants, c.1301G>A (p.Cys434Tyr), had been previously identified in a proband with multiple cutaneous and uterine leiomyomas and a history of a first-degree relative who had undergone myomectomy for uterine fibroids (10).

In a cohort of 41 patients with 46 PCC/PGLs, a 28-year-old female with an FH-deficient retroperitoneal PGL was found to have a germline *FH* variant (2.4%). Whether she had manifestations associated with HLRCC is unknown, and the causative germline *FH* variant was not specified (11).

Using LC/MS/MS, 395 PCC/PGLs from 391 patients were screened for metabolites, causing Krebs cycle alterations (12). Multigene panel sequencing was applied to detect driver pathogenic variants in cases with indicative metabolite profiles but undetermined genetic drivers. In three patients with aberrant tumor fumarate:malate, next-generation sequencing (NGS) revealed heterozygous likely pathogenic germline *FH* variants (0.8%). All three patients had unilateral adrenal PCCs with a noradrenergic biochemical phenotype and without metastatic disease. The father of a patient with an *FH* mutation, c.908T>C (p.Leu303Ser), had a history of RCC of unknown subtype. A patient with *FH* variant, c.816_836del (p.Ala273_Val279del), was diagnosed with a piloleiomyoma on her shoulder 5 years after PCC removal (12).

A recent case report described a 60-year-old woman with a previous hysterectomy for uterine fibroids, who presented with a right renal mass suspicious for RCC and an ipsilateral adrenal mass (13). Urine normetanephrine was elevated, but metanephrine was normal. After adrenalectomy and partial nephrectomy, pathology showed a low-grade RCC and a 6.8-cm PCC. Genetic testing revealed a pathogenic germline *FH* variant, which although not specified, confirmed HLRCC (13).

In a cohort of 319 patients with PCC/PGLs, two individuals with pathogenic *FH* variants were found using next-generation sequencing (0.6%). A germline variant, c.817G>A (p.Ala273Thr), was identified in a patient with a PGL and family history of PGL, and a mosaic variant, c.206G>A (p.Gly69Asp), was detected in a patient with a PCC and uterine leiomyomas (14).

A retrospective study that included 57 HLRCC patients from 38 families with 27 unique pathogenic or likely pathogenic *FH* variants described a patient with a PGL that was resected during RCC removal. Although the specific *FH* variant found on this individual was not spelled out, none of the 27 variants reported in these patients as a group was that found on our patient (15).

A large cohort of patients receiving genetic testing were included in a study to clarify the link between germline *FH* variants, HLRCC and PCC/PGL (16). Among the 909 individuals with PCC/PGL without any other genetic abnormalities, 28 had *FH* variants for a prevalence of 3.1%. When the authors classified *FH* variants *a priori* into four groups, HLRCC, fumarase deficiency, PCC/PGL variants and variant of unknown significance (VUS), patients with known HLRCC variants (1 of 290) did not have a higher prevalence of PCC/PGL compared to negative testing (0.3 vs 0.9%, respectively, *P* = 0.35). The only patient with an *FH* variant, HLRCC and PCC/PGL had a deletion of the entire coding sequence (16). However, when they interrogated the database with the *a priori* defined PCC/PGL *FH* variant category (that included the 10 previously reported *FH* variants associated with PGL/PCC), 22.2% had PCC/PGL compared to 0.9% for the group with negative testing (*P* < 0.0001). The authors concluded that some *FH* variants confer a higher risk of PCC/PGL, but not necessarily HLRCC (16). It is important to emphasize that 13 PCC/PGL patients had VUS, and it is therefore unclear if these variants were the culprit and that six patients had the *FH* variant, c.1431_1433dup (p.Lys477dup), which is pathogenic in the homozygous or compound heterozygous state for fumarase deficiency, but it is unclear if it is pathogenic in the heterozygous state for HLRCC.

In a cohort of 589 patients with PCC/PGL that underwent IHC screening for FH and/or 2SC, eight tumors (1.4%), four PCCs and four PGLs, were found to be FH-deficient (17). The four tumors with biochemical data were noradrenergic. Two PGLs were metastatic, one on presentation and the other one at 10 years. Germline testing in seven of these individuals revealed that six of them had *FH*-missense variants (17). None were known to have personal or family history of HLRCC or develop manifestations of this condition at extended follow-up (mean, 96 months; median, 54 months; and range 3–370 months). However, the mother of a 30-year-old man, with unilateral PCC and metachronous PGL diagnosed 22 years later, had a large uterine leiomyoma removed in her 30s and his sister had a PGL. Both were subsequently found to carry the same *FH* variant, c.1142C>T (p.Thr381Ile) (17).

A 64-year-old woman with end-stage renal disease was found to have a right renal mass, a left adrenal mass and a right adnexal mass on the CT scan for the evaluation of renal transplant. Biochemical and pathological examination were consistent with RCC, PCC and fallopian tube leiomyoma, respectively, strongly suggesting HLRCC. The diagnosis was, however, not confirmed as genetic testing was not performed (18).

The review of the existing literature reveals that 0.6–3.1% of individuals with PCC/PGL carry a germline variant in the *FH* gene. Among the 53 individuals with PCC/PGL and germline *FH* variants reported since 2013 (three variants were not disclosed), there were 10 individuals with PCC/PGL and personal or family history of HLRCC-related conditions. One more patient, a male diagnosed with a PCC at age 6 years, with no personal or family history of HLRCC-related conditions, was found to have an *FH* variant, c.1301G>A (p.Cys434Tyr), that had previously been detected in a woman with multiple cutaneous and uterine leiomyomas and family history of a first-degree relative who had undergone myomectomy for uterine fibroids before age 35 years (Table 1).

**Table 1:** Patients with PCC/PGL and personal and/or family history of HLRCC-related findings.

PT ID	Gender	PCC/PGL	HLRCC-related findings	Family history	DNA change	Protein change	ClinVar^‡^	Reference
1	F	PCC	Total hysterectomy for hemorrhagic fibroma at age 35 years	No	c.349G>C*	p.Ala117Pro	P/LP	Letouzé *et al.* (8)
2	F	PCC	Cutaneous piloleiomyoma noted 5 years after adrenalectomy	No	c.816_836del	p.Ala273_Val279del	P/LP	Richter *et al.* (12)
3	F	PCC	No	Father with RCC of unknown subtype	c.908T>C	p.Leu303Ser	CCP	Richter *et al.* (12)
4	F	PCC	Hysterectomy for uterine fibroids	No	NS	NS		Reda *et al.* (13)
5	F	PCC	Uterine leiomyoma and RCC	No	c.206G>A	p.Gly69Asp	VUS	Ma *et al.* (14)
6	NS	PGL	RCC	No	NS	NS		Scharnitz *et al.* (15)
7	NS	NS	HLRCC pathogenic variant	Unknown	Deletion of entire	Coding sequence	P	Zavoshi *et al.* (16)
8	M	PCC + PGL	No	Mother had a hysterectomy in her 30s for a large uterine leiomyoma and sister had a PGL. Both shared the same mutation	c.1142C>T	p.Thr381Ile	U	Fuchs *et al.* (17)
9	F	PCC	Synchronous clear cell RCC	No	c.1142C>T	p.Thr381Ile	U	Fuchs *et al.* (17)
10	FtM	PCC	Cutaneous and uterine leiomyomas and tubulocystic RCC	Mom died from uterine cancer	c.1457C>A	p.Ala486Asp	P	Present case
11	M	PCC	No	No	c.1301G>A^†^	p.Cys434Tyr	P/LP	Clark *et al.* (10)

HLRCC, hereditary leiomyomatosis and renal cell cancer; NS, not specified; PCC, pheochromocytoma; PGL, paraganglioma; PT, patient.

* This variant has been described in patients with HLRCC.

† This variant was reported in a woman with multiple cutaneous and uterine leiomyomas and a first-degree relative with uterine fibroids before age 35 years.

‡ P, pathogenic; LP, likely pathogenic; CCP, conflicting classifications of pathogenicity; U, unknown; VUS, variant of unknown significance.

Some *FH* variants have only been described in patients with isolated PCC/PGL, including c.700A>G (p.Thr234Ala) in seven patients and c580G>A (p.Ala194Thr) in three patients. Similarly, other *FH* variants have been found in patients with both isolated PCC/PGL and those with PCC/PGL and personal and/or family history of HLRCC-related manifestations, including c.1142C>T (p.Thr381Ile) in four patients and c.908T>C (p.Leu303Ser) and c.1457C>A (p.Ala486Asp) in two patients each.

Some *FH* variants, however, have been described in patients with PCC/PGL and personal and/or family history of HLRCC-related manifestations, but not in those with isolated PCC/PGL, including c.349G>C (p.Ala117Pro), c.816_836del (p.Ala273_Val279del) and c.206G>A (p.Gly69Asp), and deletion of the entire coding sequence in one patient each. Our patient’s *FH* variant, c.1457C>A (p.Ala486Asp), had been previously reported in three relatives with HLRCC, but none of them developed PCC/PGL during follow-up.

In conclusion, it has been thought that there are two different categories of patients with germline P/LP *FH* variants, those with isolated PCC/PGL and those with HLRCC and PCC/PGL, and that they do not often crossover. However, with the publication of our case report and a few other cases found after an extensive literature review, we show that there can be, indeed, crossover with HLRCC and PCC/PGL. Most PCC/PGL patients with germline *FH* variants belong to retrospective cohorts of patients in whom HLRCC was not suspected before they were recruited, examined and tested; therefore, typical clinical manifestations might have been missed and pertinent family history may have not been collected. Similarly, patients may be lost to follow-up or may not be followed long enough, or those who are not lost to follow-up may not be screened for uterine and cutaneous leiomyomas. In addition, a history of hysterectomy for uterine fibroids may not be something that could be considered relevant given the frequency of this condition in the general population. Until more information becomes available, clinicians should be aware of the possible connection between HLRCC and PCC/PGL. In the meantime, we suggest doing a full history, physical, family history, and screen for multiple cutaneous and uterine leiomyomas and RCC when there is an *FH* variant and consider an *FH* variant in those with at least one or more of the potential manifestations of HLRCC. Screening for PCC/PGL in patients with HLRCC could potentially include a baseline whole-body MRI and plasma fractionated metanephrines.

## Declaration of interest

The authors declare that there is no conflict of interest that could be perceived as prejudicing the impartiality of the work.

## Funding

This work did not receive any specific grant from any funding agency in the public, commercial or not-for-profit sector.

## Author contribution statement

J J Orrego (endocrinologist) interviewed and examined the patient and ordered all pertinent tests, and J A Chorny (pathologist) performed all the histopathological and immunohistochemical studies of the resected specimens. Both contributed to writing the manuscript.

## Patient consent

Written informed consent was obtained from the patient for publication of this case report.
